# Charge Transfer in He^+^ − He → He(1s4l, *l* ≥ 2) − He^+^ Collisions in Intermediate Energy Range

**DOI:** 10.3390/ijms25147833

**Published:** 2024-07-17

**Authors:** Patryk Kamiński, Ryszard Drozdowski

**Affiliations:** Institute of Experimental Physics, Faculty of Mathematics, Physics and Informatics, University of Gdańsk, ul Wita Stwosza 57, 80-308 Gdańsk, Poland; ryszard.drozdowski@ug.edu.pl

**Keywords:** ion–atom collision, anticrossing spectroscopy, Stark effect, density matrix

## Abstract

The anticrossing spectra of the helium line λ1s4l D3,F−1s2p P3=447.2 nm emitted after electron capture by ${He}^{+}$ ions in ${He}^{+}-He$ collisions were measured for projectile energies of 10–29 keV. Furthermore, considering the excited states’ time evolution, the theoretical intensity functions were calculated. The electric field and density distributions of the target He atoms in the collision volume were taken into account, and by fitting the theoretical intensities to the measured ones, the post-collisional states of the charge-transferred He atoms were determined. The results indicate that for the intermediate projectile energy range, the electronic charge distributions were asymmetric, but the electric dipole moments did not change, as in the case of the target atoms excited directly in the collisions. This result shows that the Paul trap mechanism may play an important role in the charge transfer excitation in this energy range.

## 1. Introduction

The study of interactions between ions and atoms, as well as between neutral atoms, is crucial both for cognitive and practical purposes. This research is important for astrophysics, for studying the upper atmosphere, and for studying various technologies of industrial processes such as plasma warming, plasma diagnosis, and thermonuclear reactions. Although we possess extensive knowledge about individual atoms and ions, describing their mutual impact often presents challenges due to their quantum nature. To date, a complete description for any pair of such objects in the entire impact energy range has not been possible. Therefore, interactions are described based on various approximations, which are then verified by experiments that provide more detailed information, enabling the creation of better mathematical models. Due to technical limitations, experiments are typically conducted only for selected ranges of variability of analysed physical quantities. Even under extremely well-established experimental conditions, it is difficult to interpret the development of the observed phenomenon. Therefore, introducing an additional external factor which does not affect the impact but differentiates the end states of the object or objects is the only way to enable the creation of the right collision model. It is important to study simple, symmetrical ion–atom systems, such as hydrogen or helium, in the collision energy range where the probability of direct excitation is comparable to that of excitation via charge transfer.

In these types of collisions, three ranges of collision energy are distinguished, for which various models of the impact description are used. According to Janev and Presnyakov [1], the ranges can be determined more accurately through the relative velocities of the colliding objects. In this way, we can determine the low-energy limit, for which the relative velocities are much lower than the velocity of an electron on the first orbit in the Bohr hydrogen atom model $v_{B}$ equal to $2.19\cdot 10^{6}\;\frac{m}{s}$. In this range of collision energy, the approximation of the molecular orbital (MO) model is used. In the high-energy limit, the Born approximation is used. The intermediate energy range covers the energy region for which the relative velocities of colliding objects are comparable with $v_{B}$. In general, a specific impact mechanism dominates in the selected collision energy, which leads to the specific effects through which this mechanism can be identified. For example, in the collision $X^{+}-He$, where $X^{+}$ stands for an ion of an atom, the following final products can be observed:

- $\to X^{+}-{He}^{*}$, target excitation;
- $\to X^{*}-{He}^{+}$, charge transfer with projectile excitation;
- $\to X^{+}-{He}^{+}-e$, target ionisation;
- $\to X^{+*}-{He}^{*}$, both target and projectile excitation.

In this paper, only the case of X=He will be analysed. In the experiments performed, the object of interest is usually one of the resulting products. In cases (i) and (ii), the excited states of the atoms immediately after the collision are of interest. In case (iii), the momentum and energy spatial distribution of the released electrons are interesting, but they are not studied here. As it turns out, in the case of (i), in the intermediate energy range, the excited atoms of the target $\left({He}^{*}\right)$ have an electric dipole moment (EDM). This means that the excited states are not stationary states of helium atoms but a specific superposition of them. These states can be determined using anticrossing spectroscopy [2,3,4]. This technique involves observing changes in the intensity of a selected spectral line as a function of the external axial electric field in the collision region. The weak external electric field does not affect the collision process but has a strong effect on the arrangement of the energy levels of He atoms and the intensity of the selected spectral lines. In the Stark effect, both the singlet and triplet energy levels shift, which can lead to their crossing or anticrossing if they are in coupled states through spin–orbit interaction [5]. For the value of the electric field intensity at which the singlet and triplet levels are anticrossed, there is a complete mixing of the wave functions of these two states. If these states are populated unequally, then the effect is a change in the intensity of the spectral lines for the transitions from these states. The intensity of the line for the transition from the more populated state decreases, and for the transition from the less populated state, it increases. We observed the characteristic peaks of the anticrossing levels, the amplitudes of which depend on the relative populations of these states. It should be added that the population of the Stark states also depends on the direction of the electric field relative to the direction of motion of the projectile ion beam. This effect is due to the EDM of the helium atom induced by the external electric field. If the EDM induced by the collision is consistent with the EDM of a particular Stark state, then this state is populated with high probability. Thus, for a given collision energy, the so-called anticrossing spectrum is recorded (i.e., the intensity of a selected singlet or triplet line as a function of the electric field in the range $\left|F\right|\leq F_{0}$, where $F_{0}$ is the fitted intensity limit to record the maximum possible number of anticrossing peaks for a given configuration). We consider the electric field to be positive or negative when it is parallel or antiparallel to the projectile velocity, respectively. The asymmetry in the intensity of the spectral line indicates that the atom has an EDM due to an asymmetric distribution of the electron cloud around the nucleus. On the other hand, the relative amplitudes of the anticrossing peaks represent the states’ relative populations. Finally, the excited states are determined by fitting the theoretically calculated anticrossing spectra to the recorded ones. In this way, the recorded anticrossing spectrum for the singlet or triplet line gives information about the populations of both states. In the case of recording a triplet line, information about the excited singlet states is contained only in the anticrossing peaks of the levels Λ1×Λ′3, where $\Lambda \equiv \left|M_{L}\right|$. For the *1s4d* configuration, there are peaks with $\Lambda ,\Lambda^{\prime }=0,1,2$ marked as A, B, C, D, and E in [5,6,7]. However, peak E (Δ1×Π)3 is the only peak which contains information about the singlet Δ state, but it occurs for an electric field strength of 181.906 ${kVcm}^{-1}$, which is far beyond the available conditions of our experimental system. Therefore, the population of the state ^1^Δ is not included in the present results, and the adopted collision mechanism indicates the excited states to be included in the theoretical calculations for a certain energy range.

To describe the collision process $X^{+}-He\to X^{+}-{He}^{*}$ leading to the excitation of the target atoms in the intermediate energy region, it has been proven to be helpful to apply the saddle dynamics [8], taking into account the Paul trap mechanism [9]. In the case of ${He}^{+}-He$ $\left(H^{+}-He\right)$ collisions in the close approach, we consider a three-body system where in the two-centre Coulomb potential generated by the projectile ion and the ${He}^{+}$ core of the target atom, the second electron of the target atom moves. The two-centre Coulomb potential has a saddle point on the axis between these centres. When one of the electrons of the target atom is on the saddle of the potential, it can stay there for a rather short amount of time since the system is in non-permanent equilibrium. However, during the relative motion of the two centres of potential, the axis of the system with the saddle point rotates. It turns out that for intermediate energies, the velocity ω of the rotation of the system axis leads to stabilisation of the electron in the saddle of the potential during the collision. In addition, the electron undergoes forced oscillations transverse to this axis [9] (Paul trap mechanism [10]). This leads to the excitation of a superposition of the stationary states 1snlL,ML1,3 of both target atoms and the resulting projectile atoms due to electron capture. These states are approximately equal to the Stark parabolic states $\left|\left.n;\;n_{1},\;n_{2},\;m_{l}\right\rangle \right.$ with the largest (positive or negative) electric dipole moments for the target with the principal quantum number *n*. These are states with $n_{1}=0$ or $n_{2}=0$ [11]. In the intermediate energy range, some measurements have been made, and collisionally excited states have been determined for He atoms as target atoms in collisions with ions and for He atoms as projectiles:

- $H^{+}-He\to H^{+}-{He}^{*}$ [12,13,14];
- ${He}^{+}-He\to {He}^{+}-{He}^{*}$ [7,15,16,17,18];
- Ar+q40−He→Ar+q40−He* [6], where q=6,13,14 describes the ionisation of argon atoms;
- $He-He\to He-{He}^{*}$ [19].

In addition, theoretical and experimental research has been published on the cross-sections for excitation of different states of target atoms as a function of collision energy with protons [20,21] and ${He}^{+}$ ions [22,23,24]. In addition, regarding cases (ii) and (iii), some papers have been published on measurement of the cross-sections for charge transfer and ionisation [21,25]. In [26], the authors collected data from many publications, and the ninth-degree polynomials were determined for the values of these cross-sections as a function of the collision energy. While the work also includes references to many papers on these processes, it analyses the total cross-sections necessary for charge transfer to the ${He}^{+}$ ion without determining the resulting excited states. Only [27,28] and [21] present the experimental results of the cross-sections for excitation of the selected states of a He atom as a result of electron capture. Case (iii) has also been repeatedly studied in the context of observations of released electrons. The idea that an important mechanism of ionisation during collisions in the intermediate energy range is the saddle point mechanism has been applied in theoretical works (for p−H [29,30,31,32,33,34,35,36,37]; p−He [38]; $A^{Y+}-B\to A^{\left(Y-r\right)+}+B^{\left(r+nq\right)+}+ne$ [39]; and $p,\;{He}^{++}-H$ [40]) and studied in experimental works ($H^{+}-H$ [41,42]; ${He}^{++}-He,H_{2}$ [43]; $C^{6+}-He$ [44]; p−He [45,46] (15 keV); ${He}^{+}-He$, ${He}^{++}-He$ [47]; ${Ne}^{+}-Ne$ [48]; and $F^{9+},I^{23+}-He$ [49]).

Although many papers have been published on charge transfer collisions, the projectiles were mainly bare atomic nuclei or multiple ionised atoms. It can be assumed in such cases that the electron of the target atom is the active electron and the excitation of projectile ions is rather unlikely, which rules out case (iv). A search of the literature reveals that only our previous paper [50] presented a method and preliminary results for determining the states of a helium atom immediately after electron capture by a helium ion with 26 keV of collision energy.

## 2. Results and Discussion

### 2.1. Experimental Set-Up

**Figure 1:**
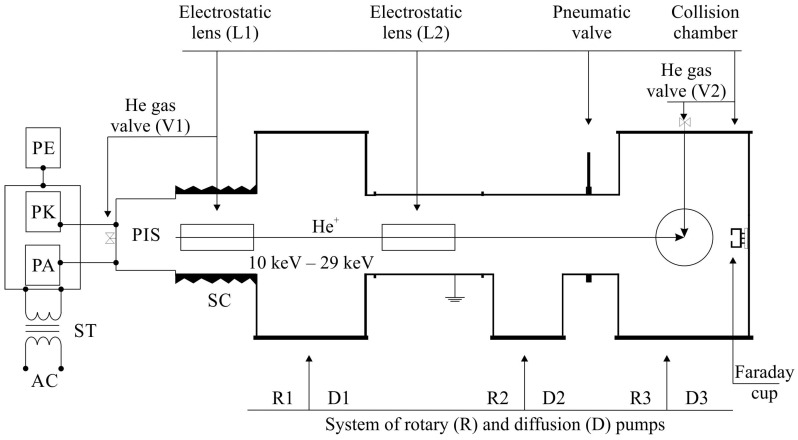
Experimental setup. The diagram of the linear accelerator and collision chamber shows PIS as the peening ion source, PK and PA as the power supplies of the anode and cathode of the PIS, respectively, and PE as the accelerator power supply determining the ion energy. More details are provided in the text.

**Figure 2:**
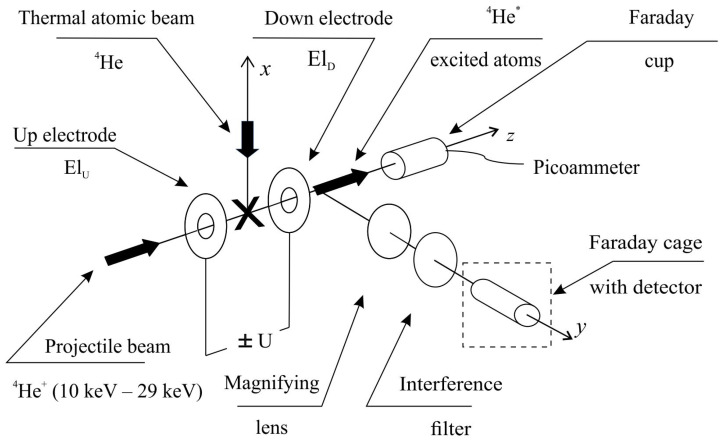
Scheme of the collision chamber and the detection system.

The observation of collisions of types (i) and (ii) involves the registration and analysis of photons emitted after the collision by the excited He atom. The same experimental set-up can be used in both cases and has been described in [6,15,16,19,50]. However, to fully understand the experimental procedure and modifications made in the detection system, it is necessary to provide important system details (Figure 1 and Figure 2).

Two separate vacuum systems: the linear accelerator and collision chamber, are divided by a pneumatic valve. The linear accelerator contains a ${He}^{+}$ ion source and an electrostatic lens system with some diaphragms to form an ion beam centred in the collision chamber. Helium is introduced to the Penning-type ion source (PIS) through a precision valve V1 and a glass separation tube, with the pressure in the tube being slightly higher than 1 bar. The PK and PA power supplies are connected to the anode (about 1 kV) and cathode (400–800 V) of the PIS, respectively, and they are powered by a 230 V AC network through an ST isolation transformer (Figure 1). The energy of the ion beam is determined by the electric potential maintained by the PE power supply as the kinetic energy qU. The PE power supply and SC separation ceramics used in the experimental set-up allow for ion energies of up to 30 keV. After the ion source, there are two electrostatic lenses (L1 and L2) which allow for proper focusing of the ion beam. The electrical potential of the lenses is to the order of 10–12 kV. In the accelerator part with an active ion beam, a vacuum of $10^{-3}$ Pa was maintained by a system of two diffusion pumps, D1 and D2 (300 L/s), cooperating with rotary pumps R1 and R2. In the collision chamber, a thermal He atomic beam was crossed with a ${He}^{+}$ beam at its focus (Figure 1). The adequate density of the thermal beam was ensured by a V2 precision valve and a stainless-steel needle 15 mm in length and an inner diameter of 0.3 mm, with a wire (0.2 mm dia.) placed inside to reduce the gas flow rate. The outlet of the needle was blunted and shaped into a circle. It was assumed that the effused thermal atomic beam had a cone shape. The outflowing atoms were directed to the diffusion pump D3 (300 L/s), which worked in conjunction with the rotary pump R3. During measurements, the pressure measured about 15 cm from the needle outlet was roughly $\left(1.1÷1.3\right)\cdot 10^{-3}$ Pa. When no gas was supplied, the pressure in both parts of the set-up was approximately $\left(2÷5\right)\cdot 10^{-4}$ Pa.

To perform measurements using the anticrossing method, the beam-crossing area (Figure 2) was positioned between two cylindrical electrodes, namely ${EL}_{D}$ and ${EL}_{U}$ (downstream and upstream electrodes, respectively). These electrodes had an outer diameter of 12 mm and an inner diameter of 3 mm. The distance between the electrodes was 3 mm, and the needle outlet was situated about 3.0 mm above the top edge of the electrode’s inner hole. Voltages of opposite polarity, ranging from −6 kV to +6 kV, were applied to the electrodes using Heinzinger power supplies (HNC—10000, Heinzinger electronic GmbH, Rosenheim Germany) controlled by a computer, resulting in an axial electric field of ±30 ${kVcm}^{-1}$ in the centre between the electrodes. The electric field distribution in the collision volume was dependent on these details.

Considering the excitation of thermal atoms of the target beam, it could be assumed that all excited He target atoms, due to the short lifetime of the 1snl levels (n=4 and l=1, 2, 3), made the transition to states with lower energy, relatively instantly radiating a quantum of light. (For example, for τ(4D)3=32 ns, the displacement of thermal atoms at 300 K during their lifetime is $4\cdot 10^{-2}\;mm$.) In contrast, a ${He}^{+}$ ion with kinetic energy of 30 keV moves at a speed of $1.2\cdot 10^{6}\;\frac{m}{s}\approx 0.55v_{0}$. Taking into account the small excitation energy of the He atom compared with the kinetic energy of the ion, the excited He (4D)3 atom formed after electron capture travelled a distance of 38 mm. This means that the probability of photon emission by a fast atom in the collision region between electrodes was small. It can be estimated that for the above conditions, more than 95% of fast atoms excited to the 4D3 state radiated spontaneously in the area outside the electrodes. Thus, in such an arrangement, the radiation of thermal atoms recorded between the electrodes is only slightly disturbed by the radiation of fast atoms. At the same time, the effective registration of the radiation of fast atoms requires a much larger observation area. By using appropriate diaphragms and a lens, the area from 12 mm to 22 mm from the centre collision zone was imaged on the cathode of a Hamamatsu R 2257 photomultiplier operating in its single-photon counting mode (HAMAMATSU PHOTONICS, Hamamatsu City, Japan). The photomultiplier was cooled to −30 °C using a Hamamatsu C 2761 cooler. The signal from the photomultiplier was amplified and processed by an SR 445 preamplifier (Stanford Research Systems, Sunnyvale CA, USA) and an SR 400 two-channel photon counter (Stanford Research Systems, Sunnyvale CA, USA.). The counter, via an IEEE-488 interface (Iotech, Inc., Cleveland, OH, USA), was coupled to a PC containing a PCI488 card from IOtech. An observation was carried out in the direction perpendicular to both crossing beams. To select the spectral line λ1s4l D3−1s2p P3=447.2 nm, a Knight Optics Knight Optical interference filter with FWHM=2 nm and 33% transmissivity (Knight Optical Ltd., Roebuck Business Park Harrietsham, Kent, United Kingdom) was used in front of the photomultiplier cathode. This line was thoroughly separated because only the spectral line λ1s5s S1−1s2p P1=443.9 nm was closest to it and was transmitted through this filter with an efficiency below 3%. The whole detection system was placed in a Faraday cage to eliminate external interference, as shown in Figure 2. The intensity of the ion beam was monitored by measuring the current from the Faraday cup with a Keithley 6485 picoammeter (Keithley Instruments, Inc. Cleveland, OH, USA). A suitably chosen negative potential was applied to the outer electrode of the Faraday cup so that all secondary electrons were knocked out of the centre electrode by energetic helium atoms, and the ions remained trapped. This prevented distortion of the current measurement of the ion beam as a function of its kinetic energy. The fraction of ions capturing electrons was quite small and had no effect on the current of the monitored ion beam, which in the experiment was to the order of μA ($6\times 10^{12}$ ${He}^{+}$ ions per second). In the experiment, the conditions of a single collision were preserved, and the detection system operating in single-photon counting mode recorded about a thousand photons per second in an observation area 10 mm wide.

### 2.2. Theoretical Intensity Functions

The purpose of the measurements was to analyse the process of electron capture with the ${He}^{+}$ ion from He atoms due to collisions in the intermediate energy range. This meant determining the states of He atoms formed immediately after the collision and measuring the relative cross-sections for creating atoms in specific states. After the collision, the atom may be in a state which is a superposition of stationary states with an energy $E_{k}$ [51]:

(1) $$\Psi =\sum_{k}c_{k}\left|\left.k\right\rangle \right.e^{-iE_{k}t/\hbar }$$

When the atom is in the state described by Equation (1), the electron cloud centre of the atom need not coincide with the nucleus’ centre; in such a case, the atom will have an electric dipole moment (EDM). However, the determination of the post-collision states (i.e., the determination of the $c_{k}$ coefficients) requires a special measurement technique. By measuring the intensities of the selected spectral lines, we could determine the defined cross-sections for forming excited fast atoms but not the states themselves, because the cross-sections were proportional to the squares of the $c_{k}$ coefficients. This led to a loss of information about the phase relations between excited states $\left|\left.k\right\rangle \right.$. The line intensity depends on the states’ populations involved in the observed transitions. In the electric field, when its strength changes, the populations of these states change because of the Stark effect. In this way, the excited state can be theoretically reproduced. Therefore, anticrossing spectroscopy is especially useful, whereby the spectral lines emitted by excited target atoms and emitted by excited fast He atoms can be separated, and changes in their intensities allow reproduction of the excited states in the collisions.

**Figure 3:**
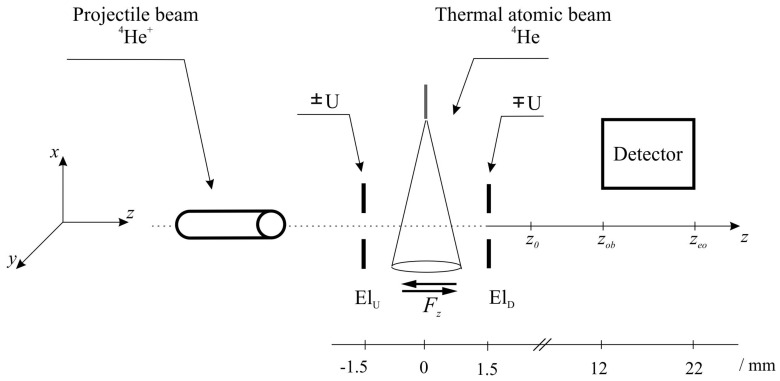
Scheme of the beams of ${He}^{+}$ ions and *He* target atoms.

In experiments involving ensembles of atoms or ions, using the time-dependent density matrix theory to describe their temporal evolution is convenient. The density matrix depends on the excitation process and external conditions (i.e., whether an electric or magnetic field is present). Thus, when taking the Z axis as the direction of motion of the helium ions, the temporal evolution of the density matrix $\rho \left(z(t),t\right)$ describes the capture of electrons by the ions (i.e., the formation of excited atoms) and their motion in the electric field and then in free space until the emission of a photon in the observation zone (Figure 3).

(2) $$\frac{d}{dt}\rho \left(z(t),t\right)={\left(\frac{d}{dt}\rho \left(z(t),t\right)\right)}_{excitation}+{\left(\frac{d}{dt}\rho \left(z(t),t\right)\right)}_{evolution}+{\left(\frac{d}{dt}\rho \left(z(t),t\right)\right)}_{decay}.$$

The formation of excited fast helium atoms (electron capture by ${He}^{+}$ ions) is described by the excitation matrix $\sigma^{ex}\left(z(t),t\right)$:

(3) $${\left(\frac{d}{dt}\rho \left(z(t),t\right)\right)}_{excitation}=\sigma^{ex}\left(z(t),t\right)$$

where the excitation matrix is written as the product of the $\sigma^{0}$ matrix describing the electron’s capture in the state $\left|k\right\rangle$, regardless of the spatial size of the intersecting projectile and target beams, and the distribution of the “probability” $g\left(z\right)$ of the ion meeting the target atom, which is proportional to the area of the beam intersection:

(4) $$\sigma^{ex}\left(z\left(t\right),t\right)=g\left(z\right)\sigma^{0}.$$

The diagonal elements of the $\sigma^{0}$ matrix describe the average number of atoms excited to defined states per unit of time when colliding beams with defined densities and energies cross. Denoting the intensity of the ion flux as *I*, the probability of excitation of a specific state $\left|k\right\rangle$ can be defined as:

(5) $$P_{k}=\frac{\sigma_{kk}^{0}}{Tr\sigma^{0}}=\rho_{kk}=\frac{I\cdot \sigma_{kk}^{0}}{Tr\left(I\cdot \sigma^{0}\right)}=\frac{\sigma_{k}^{0}\left(\left|k\right\rangle \right)}{\sum_{i}\sigma_{i}^{0}\left(\left|i\right\rangle \right)}=\sigma \left(\left|k\right\rangle \right),$$

where $\sigma_{k}^{0}\left(\left|k\right\rangle \right)=I\cdot \sigma_{kk}^{0}$ is the total cross-section for the formation of the $\left|k\right\rangle$ state. The diagonal elements of the density matrix are proportional to the diagonal elements of the excitation matrix through the normalisation factor $Tr\sigma^{0}$. Naturally, the probability $P_{k}$ is also given by the relative cross-sections for the formation of the $\left|k\right\rangle$ state, denoted as $\sigma \left(\left|k\right\rangle \right)$ for simplicity. For calculations, we arbitrarily assumed that $Tr\sigma^{0}=100$.

**Figure 4:**
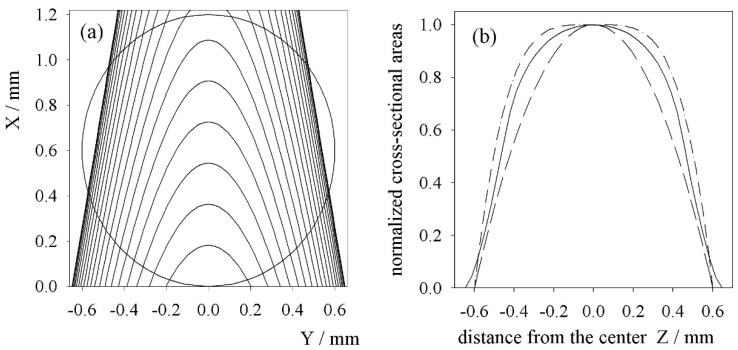
(**a**) A geometrical cross-section of an ion beam in the form of a cylinder with a radius $r_{i}=0.6$ mm with a conical beam of atoms with a radius $r_{z}=0.54$ mm in the plane of the Z axis. (**b**) The normalised cross-sectional areas. The solid line represents the cross-sections calculated numerically from Figure 4a. The dashed line and the dot-dashed line represent analytical functions given by Equation (6) with *n* = 2 and 4, respectively.

The ion beam was assumed to be a cylinder in which the ions were uniformly distributed, and the beam of target atoms formed a cone (the radius of the ion beam $r_{i}=0.6$ mm, and the radius of the cone of the beam of atoms on the Z axis $r_{z}=0.54$ mm [17]). Figure 4a shows the cross-sections of these two geometric figures.

It can be seen from this that making the assumption that ${Tr\sigma }^{0}=100$ implies the formation of 100 excited fast atoms to the defined states in a layer of $r_{i}^{2}dz$ per second. This type of consideration does not delve into the processes that lead to certain states. In Figure 4b, the solid black line illustrates the normalised geometrical cross-sections calculated numerically from Figure 4a, and the other lines illustrate analytical functions which can be used as an approximation of the distribution of the excitation function. The function $g\left(z\right)$ takes the form

(6) $$g\left(z\right)=1-{\left(\frac{z-r_{z}}{r_{z}}\right)}^{2n}\;\mathrm{for}\;\left|z-r_{z}\right|\leq r_{z},\;g\left(z\right)=0\;\mathrm{for}\;\left|z-r_{z}\right|>r_{z},$$

In the calculations, we finally assumed that n=2. Moreover, the calculations show that a change in the density of the target atoms in the beam has no significant effect on the results of the calculations. In addition, it was assumed that all target atoms were in the ground state before the collision and that cascading repopulations were negligible. After the capture of electrons by ${He}^{+}$ ions, a significant portion of the resulting excited atoms move toward the Faraday cup, and the Liouville equation describes their temporal evolution:

(7) $${\left(\frac{d}{dt}\rho \left(z(t),t\right)\right)}_{evolution}=-\frac{i}{\hbar }\left[H\left(z(t),t\right),\rho \left(z(t),t\right)\right],$$

where $H\left(z\left(t\right),t\right),$ is the Hamiltonian of excited He atoms in an electric field and square brackets denote the commutator. We assumed that, under single collision conditions, the loss of excitation energy occurred through the emission of light quanta, and the change in states as a result of such a process is described by the anti-commutator (square brackets with a subscript +):

(8) $${\left(\frac{d}{dt}\rho \left(z(t),t\right)\right)}_{decay}=-\frac{1}{2}{\left[D\left(z\right),\rho \left(z(t),t\right)\right]}_{+},$$

where the matrix *D(z)* is a diagonal decay matrix whose elements are the decay constants of individual states.

In the stationary case (as described above), we can eliminate the time dependence from the equations:

(9) $$\frac{d}{dt}\rho \left(z(t),t\right)=\frac{dz(t)}{dt}\frac{d}{dz}\rho \left(z\right)=v\frac{d}{dz}\rho \left(z\right).$$

We can write the equation for the density matrix depending only on the position:

(10) $$v\frac{d}{dz}\rho \left(z\right)=\sigma \left(z\right)-\frac{i}{\hbar }\left[H\left(z\right),\rho \left(z\right)\right]-\frac{1}{2}{\left[D\left(z\right),\rho \left(z\right)\right]}_{+}.$$

Because the density matrix is a complex matrix with the real and imaginary parts

(11) $$\rho \left(z\right)=\rho^{R}\left(z\right)+{i\rho }^{I}\left(z\right),$$

we have to solve a system of equations:

(12) $$\left\{\begin{array}{c} v\frac{d}{dz}\rho^{R}\left(z\right)=\sigma^{R}\left(z\right)+\frac{1}{\hbar }H\left(z\right)\rho^{I}\left(z\right)-\frac{1}{\hbar }\rho^{I}\left(z\right)H\left(z\right)-\frac{1}{2}D\left(z\right)\rho^{R}\left(z\right)-\frac{1}{2}\rho^{R}\left(z\right)D\left(z\right)\\ v\frac{d}{dz}\rho^{I}\left(z\right)=\sigma^{I}\left(z\right)-\frac{1}{\hbar }H\left(z\right)\rho^{R}\left(z\right)+\frac{1}{\hbar }\rho^{R}\left(z\right)H\left(z\right)-\frac{1}{2}D\left(z\right)\rho^{I}\left(z\right)-\frac{1}{2}\rho^{I}\left(z\right)D\left(z\right), \end{array}\right.$$

whereby the excitation matrix σ has a real and an imaginary part. The real part has diagonal and off-diagonal elements, and the imaginary part can only have off-diagonal elements. Furthermore, it was assumed that the elements of an imaginary part of the excitation matrix $\sigma^{0}$ are equal to zero because we thought that only the states’ populations were known after a collision:

(13) $$\sigma^{0}=\sigma^{0R}.$$

Moreover, due to the rotational symmetry of the system concerning the ion beam, only matrix elements with magnetic quantum numbers satisfying the condition $m=m^{\prime }$ can be different from zero. Furthermore, due to reflection symmetry for any plane containing the ion beam, the matrix elements do not depend on the sign of the magnetic quantum number m [52].

By using the Runge–Kutta method to integrate the system of the equations in Equation (12), the diagonal elements of the density matrix (representing state populations) were determined:

(14) $$P_{k}\left(z\right)=\rho_{kk}\left(z\right).$$

The intensity of the emitted spectral line, expressed as the number of emitted photons in the transition from the $\left|\left.k\right\rangle \right.$ state to the $\left|\left.i\right\rangle \right.$ state, is given by the formula

(15) $$I_{ik}^{c}\left(z\right)=W_{ik}N_{k}=W_{ik}P_{k}\left(z\right)N,$$

where *W_ik_* is the transition probability between states $\left|\left.k\right\rangle \right.$ and $\left|\left.i\right\rangle \right.$, $N_{k}$ is the density of atoms in the excited state $\left|\left.k\right\rangle \right.$, and *N* is the density of all projectiles. Taking into account that the observation was carried out at the solid angle *dΩ*, we could then define the normalised intensity at the solid angle:

(16) $$I_{ik}\left(z\right)d\Omega =\frac{1}{N}\frac{dI_{ik}^{c}\left(z\right)}{d\Omega }d\Omega =\frac{dW_{ik}}{d\Omega }P_{k}\left(z\right)d\Omega ,$$

which we could count, having found the values of $P_{k}\left(z\right)$. On the other hand, the function $\frac{dW_{ik}}{d\Omega }$ determines the probability of transition of an atom from a state of energy $E_{k}$ to a state of energy $E_{i}$ with the emission of a photon with a polarisation direction $e_{j}$ at the solid angle dΩ [11]:

(17) $$\frac{dW_{ik}\left(\Omega ,j\right)}{d\Omega }=\frac{e^{2}}{hc^{3}}\omega_{ik}^{3}{\left(e_{j}\cdot r_{ik}\right)}^{2},$$

where $r_{ik}=\left\langle i|r|k\right\rangle$, $\omega_{ik}=\left(E_{k}-E_{i}\right)/\hbar$. Thus, given Equations (15) and (16), we have

(18) $$I_{ik}\left(z\right)=\frac{e^{2}}{hc^{3}}\omega_{ik}^{3}{\left(e_{j}\cdot r_{ik}\right)}^{2}P_{k}\left(z\right).$$

**Figure 5:**
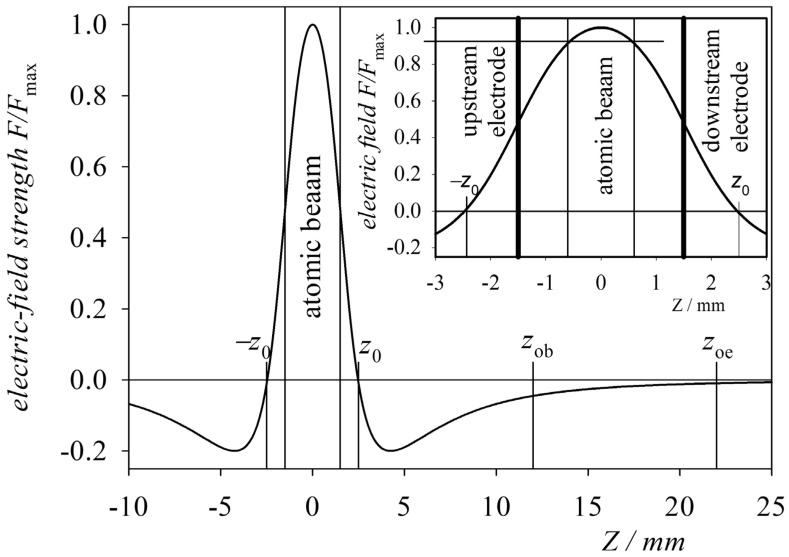
Electric field intensity distribution in the experimental system along the Z axis. The inset shows an expanded region around z=0.

**Figure 6:**
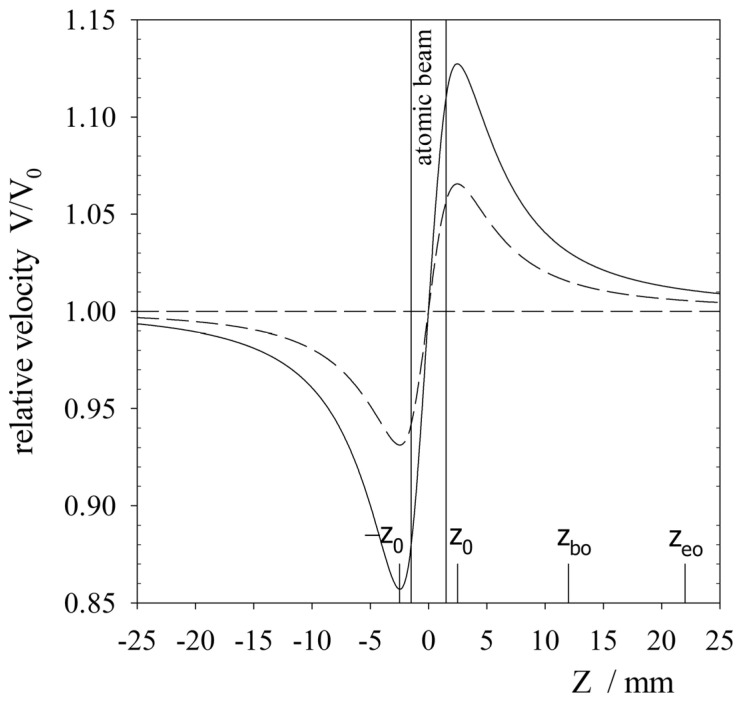
Change in velocity of the helium ions in the experimental system. The solid line is for the ion beam energy of 15 keV, and the dashed line is for the ion beam energy of 30 keV.

**Figure 7:**
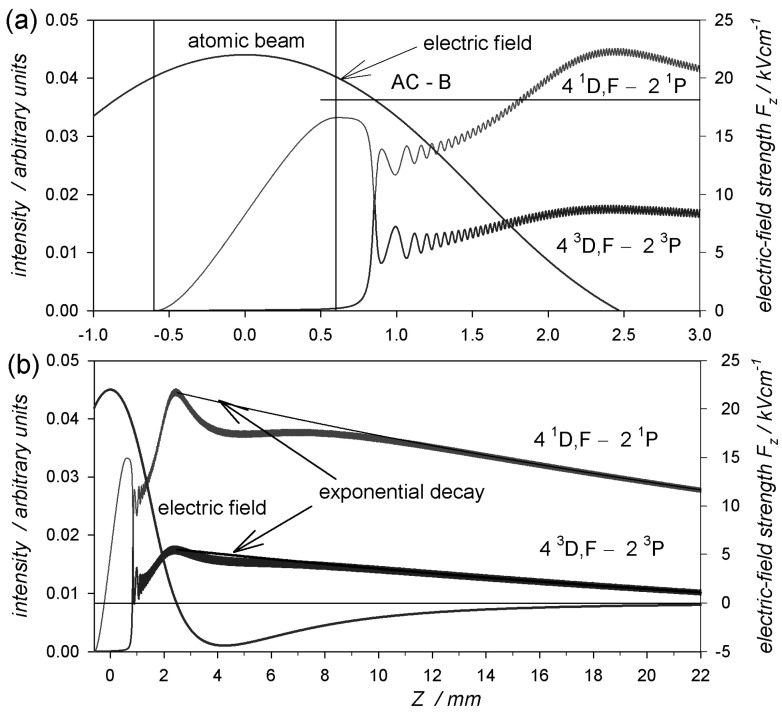
Intensities of spectral lines—singlet λ4 D1,F−2 P1=492.2 nm and triplet λ4 D3,F−2 P3=447.2 nm—along the *Z* axis (left vertical axis) and the electric field distribution (right vertical axis) for 22 ${kVcm}^{-1}$ in the midpoint between electrodes. The calculations were made for fast atoms with an energy of 26 keV $(v=1.12\cdot 10^{6}\;\frac{m}{s}$). (**a**) The area of the atomic beam and the electric field of the AC-B peak ($E_{B}\approx 18.14\;kV{cm}^{-1}$). (**b**) The calculation extended to the observation region. The curves of the exponential decays of the intensity of the spectral lines (Equation (25)), with τ=37 ns for the singlet line and τ=32 ns for the triplet line, are presented.

The observation was carried out perpendicular to the plane of the crossing beams (Figure 1) (i.e., the recorded radiation had a wave vector $k=\left|k\right|e_{k}=\left|k\right|e_{y}$). Thus, the XZ plane was perpendicular to the wave vector and contained all possible polarisation vectors of the emitted electromagnetic radiation. For unpolarised light, we obtained

(19) $$I_{ik}\left(z\right)=\frac{e^{2}}{hc^{3}}\omega_{ik}^{3}\left(z_{ik}^{2}+x_{ik}^{2}\right)P_{k}=\frac{e^{2}}{hc^{3}}\omega_{ik}^{3}\left({\left(r_{0}^{\left(1\right)}\right)}_{ik}^{2}+\frac{1}{\sqrt{2}}{\left({\left(r_{-1}^{\left(1\right)}\right)}_{ik}-{\left(r_{+1}^{\left(1\right)}\right)}_{ik}\right)}^{2}\right)P_{k}\left(z\right),$$

where $x_{ik}$ and $z_{ik}$ are the matrix elements of the Cartesian tensor operators and ${\left(r_{q}^{\left(1\right)}\right)}_{ik}$ are the matrix elements of the spherical tensor operators [11]. All calculations were performed on the spherical harmonics basis $\left\{\left|\left.\left(L,S\right)J,M_{J}\right\rangle \right.\right\}$. On the other hand, the eigenfunctions of the Hamiltonian of the helium atom were determined as performed in studies with direct excitation of the target atoms (helium atoms), assuming the one-configuration approximation for the principal quantum number n=4 [5,12]. These calculations were verified by direct comparison of the calculated He spectra with experimentally recorded spectral lines in electric fields up to 1600 ${kVcm}^{-1}$ [53]. However, it must be considered that an electric field between two circular electrodes with holes is not homogeneous. The distribution of the electric field strength calculated numerically in the experimental set-up used is presented in [16]. However, it can be considered that the ion beam moved within a field on the axis of the two-electrode system at a distance of d=3 mm due to the ratio of the width of the ion beam, which had a radius of 0.3 mm, to the radius of the circular hole ($R_{0}=1.5$ mm) and the radius of the electrodes (R=6 mm). The normalised electric field distribution between the electrodes was easily calculated analytically ($d_{\pm }=\left(\frac{d}{2}\pm z\right)$):

(20) $$F_{z}=\frac{F_{0}}{0.9291}\left[d_{+}\left(\frac{1}{\sqrt{d_{+}^{2}+R^{2}}}-\frac{1}{\sqrt{d_{+}^{2}+R_{0}^{2}}}\right)+d_{-}\left(\frac{1}{\sqrt{d_{-}^{2}+R^{2}}}-\frac{1}{\sqrt{d_{-}^{2}+R_{0}^{2}}}\right),\right]$$

where $F_{0}$ is the maximum value of the electric field at the centre between the electrodes and the normalisation factor 0.9291 is due to the system’s geometry. The distribution of the calculated axial field is shown in Figure 5. As can be seen, in the area where the beam of target atoms was located, the value of the electric field varied by less than 8% (inset in Figure 5), and we considered this field to be constant. Furthermore, the projectile’s velocity changed due to the Coulomb force in the region between the electrodes qE. If the upstream electrode (closer to the source) had a positive potential, then the ${He}^{+}$ ion approaching the electrode, having a velocity $v_{\infty }$ at infinity, was decelerated until the electric field changed signs at the point −$z_{0}$ (Figure 6). From this point, it accelerated until the electric field again changed signs at the point $z_{0}$. Further on, it decelerated again to reach the velocity $v_{\infty }$ at infinity. When the ion captured an electron and became an excited atom, it was no longer influenced by the Coulomb force and continued to move at a constant velocity obtained during the capture of the electron. Thus, fast atoms can only move with such velocities as those of ions in the region of crossing the atomic beam (the region denoted as “atomic beam” in Figure 5 and Figure 7). To calculate these velocities, the principle of energy conservation was used. Thus, the velocity of the ion is given by the expression

(21) $$v=\sqrt{v_{\infty }^{2}-\frac{2e}{m}V},$$

where *V* is the electric potential $\left(V\left(\infty \right)=0\right)$ satisfying the equation along the *Z* axis:

(22) $$F_{z}=-\frac{\partial V}{\partial z}$$

This means we have

(23) $$V\left(z\right)=V\left(\infty \right)-\oint_{\infty }^{z}Fdz^{\prime }.$$

The integration of Equation (20) is straightforward:

(24) $$V\left(z\right)=F_{nor}\left(\sqrt{d_{+}^{2}+R^{2}}-\sqrt{d_{+}^{2}+R_{0}^{2}}-\sqrt{d_{-}^{2}+R^{2}}+\sqrt{d_{-}^{2}+R_{0}^{2}}\right).$$

Figure 6 shows the velocity variations of helium ions with kinetic energies of 15 keV and 30 keV when the maximum electric field between the electrodes was 30 ${kVcm}^{-1}$. As can be seen, in the collision regions, these velocities ranged from 0.88$v_{\infty }$ to 1.11$v_{\infty }$ and from 0.95$v_{\infty }$ to 1.05$v_{\infty }$, respectively. Obviously, the higher the kinetic energy of the ion, the smaller the percentage changes in velocity. Thus, the velocity distribution of the fast atoms in the beam was concentrated around the central velocity $v_{0}=v_{\infty }.$ In addition, as can be seen in Figure 4, the interaction area of the ion beam with the conical beam of target atoms was even smaller than the assumed atomic beam width of $2r_{z}$. Therefore, it is reasonable to suppose in the calculations that the electric field did not affect the velocity of the fast helium atoms $v_{\infty }.$

After capturing electrons, helium atoms move along the Z axis to the observation zone at the speed they acquire during electron capture. This zone is denoted as $z_{bo}$–$z_{eo}$. To calculate the total intensity of the selected spectral line in the observation zone, the line intensities in this zone must be summed using Equation (19), and this formula must be integrated within given limits. The Equation (12) must be solved up to the upper limit $z_{eo}=22$ mm. This is a long distance in atomic units $a_{o}$ (the Bohr radius), and thus the calculation time is quite long, but it can be shortened through the use of some simplifications. When there is no electric field, or the field strength is weak, the spectral line’s intensity decreases exponentially. This means that with the intensity of the spectral line λ for a transition between two states $\left|\left.k\right\rangle \right.$ and $\left|\left.i\right\rangle \right.$ at point $z_{o}$ $\left(I_{ik}\left(z_{o}\right)\right)$, the intensity of this line can be calculated at any point in the observation zone $z>z_{0}$ (Figure 3):

(25) $$I_{\lambda }^{ob}\left(z\right)=I_{ik}\left(z_{o}\right)exp\left(-\frac{t-t_{0}}{\tau_{\lambda }}\right)=I_{ik}\left(z_{o}\right)exp\left(-\frac{v_{0}\left(t-t_{0}\right)}{{v_{0}\tau }_{\lambda }}\right)=I_{ik}\left(z_{o}\right)exp\left(-\frac{z-z_{0}}{l_{\tau \lambda }}\right),$$

where $\tau_{\lambda }=\frac{1}{\sum_{i}A_{ik}}$ is the lifetime of the state $\left|\left.k\right\rangle \right.$ and $A_{ik}$ is the rate of transition from state $\left|\left.k\right\rangle \right.$ to state $\left|\left.i\right\rangle \right.$ with lower energy $E_{i}$, and $l_{\tau \lambda }={v_{0}\tau }_{\lambda }$. By integrating this equation in the limit from $z_{ob}$ to $z_{oe}$, we obtained

(26) $$I_{\lambda T}=I_{ik}\left(z_{o}\right)l_{\tau \lambda }\left(exp\left(-\frac{z_{ob}-z_{0}}{l_{\tau \lambda }}\right)-exp\left(-\frac{z_{oe}-z_{0}}{l_{\tau \lambda }}\right)\right).$$

Obviously, $I_{ik}\left(z_{0}\right)$ depends on the excitation matrix, and for the given experimental conditions, the values of the matrix elements depend on the densities of the crossing beams. Therefore, we used relative units in the calculations, and to make these units convenient for Equation (25), a factor of $10^{4}$ was introduced:

(27) $$I_{\lambda }=10^{4}\cdot I_{\lambda T}.$$

To determine the excited states with n=4, l≥2 we used the spectral line I4471s4l D3 → 1s2p P3=447.2 nm. The states 1s4 DJ3 with J=1, 2, 3 and 1s4 FJ3 with J=2, 3, 4 lay extremely close to each other, with the energy difference being only about 7.4 cm^−1^. Similarly, for the states 1s4 D21 and 1s4 F31 , we had an energy difference of 5.44 cm^−1^ [5,6]. If there were no electric field, then the 1s4 F1,3 → 1s2p P1,3 transitions would be forbidden. However, the electric field mixed states of opposite parity, and then these singlet and triplet lines appeared. For appropriate values for the electric field, anticrossing of the energy levels of the singlet and triplet configurations $\left|\left.1s4l\;\right\rangle \right.$ occurs [5,7]. These affect the populations of states and the intensity of the recorded spectral line $I_{447}$.

To illustrate the above considerations, Figure 7 shows the calculated intensities of two spectral lines, namely the singlet λ1s4l D1,F−1s2p P1=492.2 nm and the triplet λ1s4l D3,F−1s2p P3=447.2 nm, as a function of the distance along the *Z* axis. The fast atoms moved at a velocity of $v=1.12\cdot 10^{6}\;\frac{m}{s}$ (26 keV, the kinetic energy). To additionally present the mechanism of the formation of anticrossing peaks, it was assumed that the singlet parabolic state 4,0,3  Σ1 (more details are provided later in this paper) was excited. The calculations were carried out for the maximum electric field value between the electrodes, being equal to 22 ${kVcm}^{-1}$.

In the area marked as the atomic beam in Figure 7a, from −0.6 mm to 0.6 mm, ${He}^{+}$ ions, capturing electrons, transferred to the excited singlet state of the He atoms, and the intensity of the singlet line increased, but the intensity of the triplet line was equal to zero. This process occurs in an electric field with a value greater than 18.14 kVcm^−1^ (i.e., the value for which the anticrossing peak AC-B is observed) [7]. However, the electric field along the *Z* axis decreased, and when *Z* equaled about 0.855 mm, it reached the value of the AC-B peak in the case under consideration. It was already outside the area of the atomic beam. In this region, the coupled singlet and triplet states were completely mixed. This resulted in the appearance of a triplet line and small oscillations in the intensities of both lines. The oscillation period was proportional to the energy difference in the coupled states (in this case, it yielded ∼0.019 mm on the *Z* axis). Further evolution of these states took place in the electric field lower than $E\left(AC-B\right)$. The intensities of both lines reached their maximum for E=0 at $z_{0}=2.5$ mm (Figure 7). We took the values of these maxima as the corresponding $I_{ik}\left(z_{o}\right)$ amplitude in Equation (25). In Figure 7b, the exponential decay curves of the intensity of these lines are marked as “exponential decay”, in which the lifetime τ=37 ns for the singlet state and τ=32 ns for the triplet state were assumed. As can be seen, these curves coincide quite precisely with the direct calculations of the intensities in the observation region from 12 mm to 22 mm, which justifies the use of Equation (25). We checked many values for the electric field and various excited states, and we found that the small deviations in the exponential decay from accurate calculations did not affect the intensity in the observation area.

### 2.3. Calculations

The procedure for determining the excited states of fast helium atoms resulting from electron capture is the same as that used for excited target atoms [15,16,19]. First, the base anticrossing spectra were calculated (i.e., the intensity of the spectral line (Equation (26)) as a function of the electric field strength from −30 ${kVcm}^{-1}$ to 30 ${kVcm}^{-1}$), assuming that a certain state was excited.

**Figure 8:**
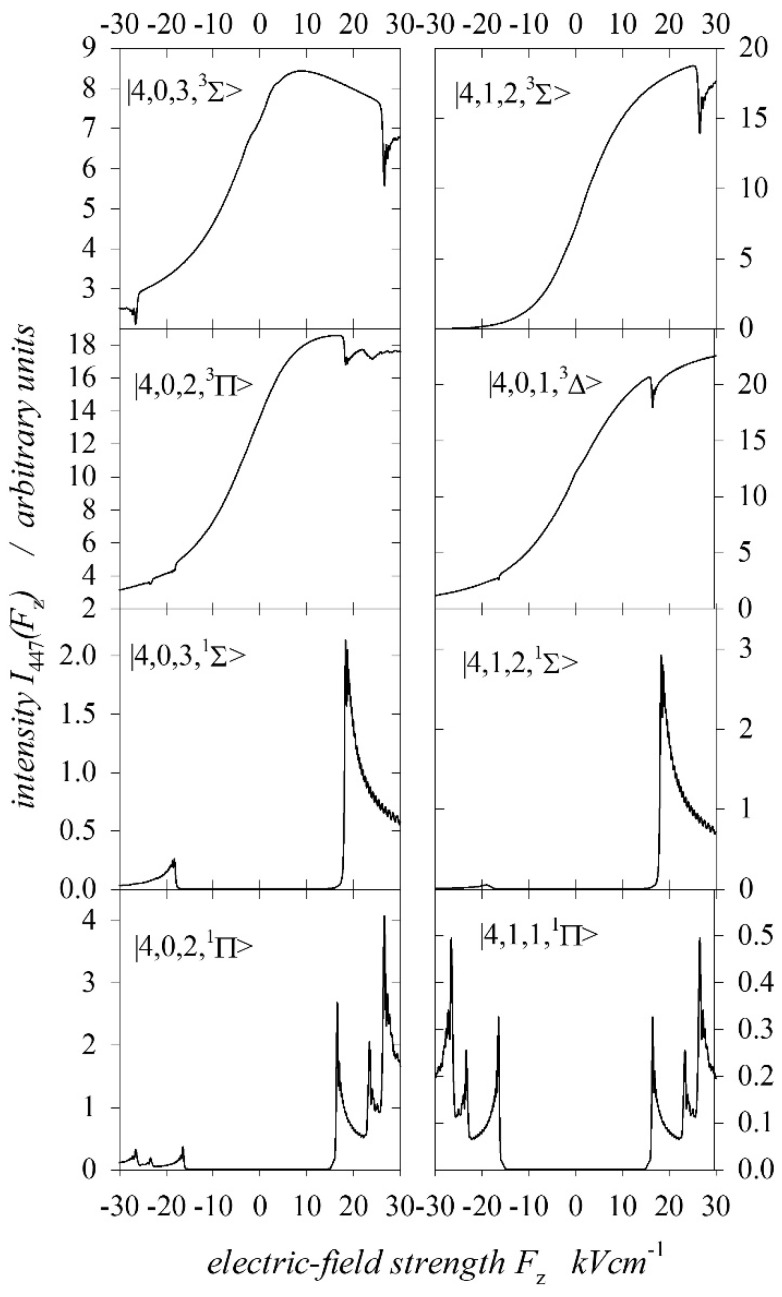
Intensity functions $I_{447}$ calculated for selective excitation of parabolic singlet Σ,Π and triplet Σ,Π,Δ states for 29 keV of ${He}^{+}$ energy.

**Figure 9:**
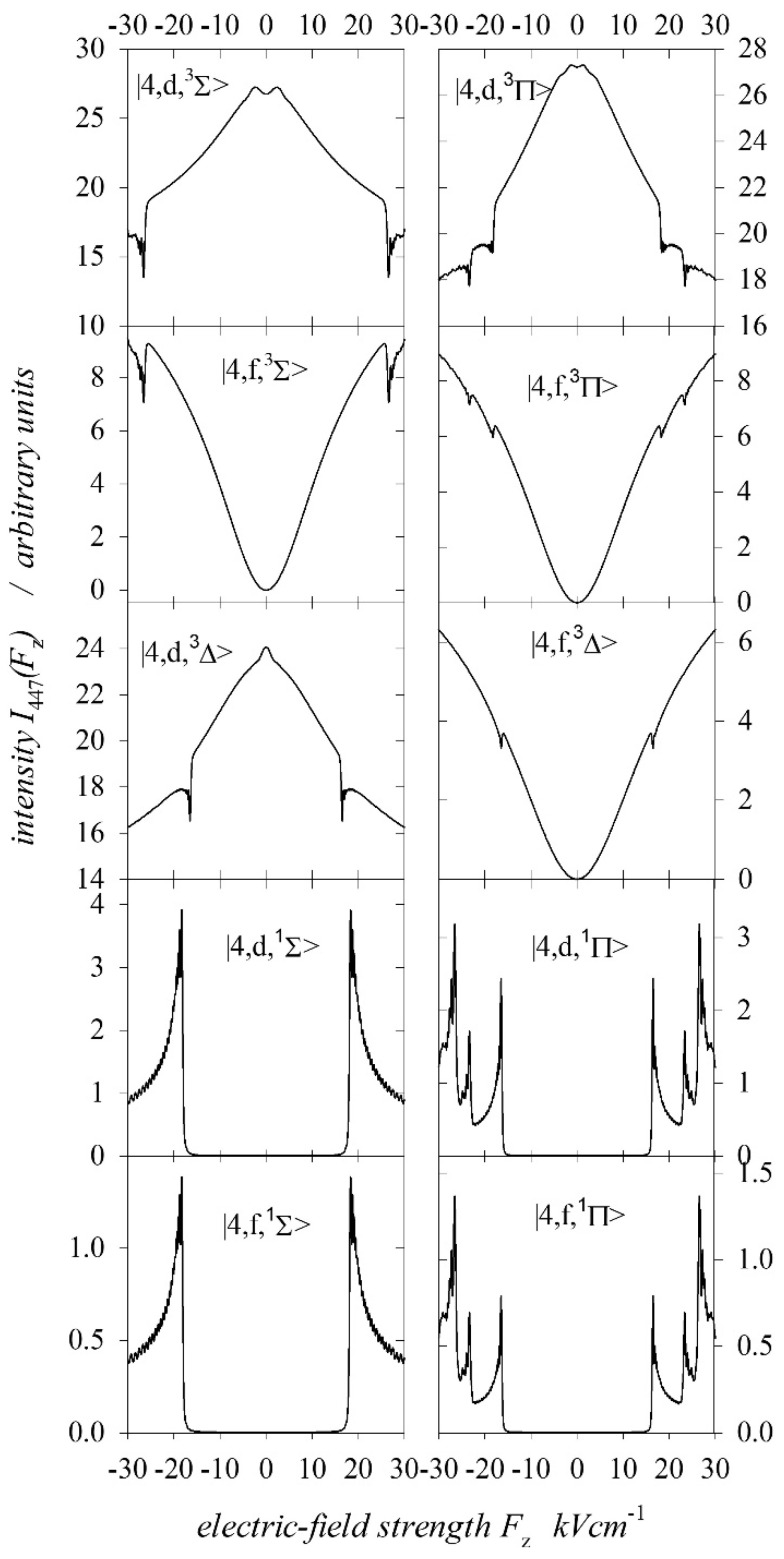
Intensity functions *I*_447_ calculated for selective excitation of spherical triplet and singlet states 1s4l Λ3,1Λ=0,1,2 for 29 keV of ${He}^{+}$ energy as a function of the external electric field.

As mentioned earlier, previous research suggests that the Paul trap mechanism is important for the formation of excited target helium atoms. This mechanism leads to the formation of states with an EDM, and we predicted that this was also the case for the charge transfer states. Therefore, in such cases, the optimal bases of states are the parabolic states $\left|\left.n,n_{1},n_{2},m_{L}\;\right\rangle \right.$ [11], whose EDM is given by the formula

(28) $$d_{z}=-\frac{3}{2}n\left(n_{1}-n_{2}\right)ea_{o},$$

where $n_{1}$ and $n_{2}$ are parabolic quantum numbers. In an electric field, the states degenerate due to the sign of the magnetic orbital quantum number, which plays the most important role in the Stark effect. Therefore, we will denote parabolic states together with spin as n,n1,n2,mL S≡n,n1,n2,Λ2S+1 , with $\Lambda =\left|m_{L}\right|$ and S=0, 1 for the singlet and triplet states, respectively. In the case of triplet states, this state represents the sum of three states, with $m_{S}=0,\;\pm 1$. The direction of the EDM vector of these states should be the opposite of that of the excited target atoms. Therefore, only states with large EDM values were considered (i.e., with $n_{1}=0,\;1$). For example, Figure 8 shows the calculated intensities of the $I_{447}$ line for the excited parabolic triplet and singlet states for a ${He}^{+}$ ion energy of 29 keV, and it includes only the calculation results which illustrate the excited states necessary for determining the states of fast atoms. For these parabolic states, the line intensities of $I_{447}$ were characterised by a quite large asymmetry with respect to the direction of the axial electric field. This was also true for the amplitudes of the characteristic anticrossing peaks. In the case of excitation of spherical states, the intensity of the spectral line $I_{447}$ did not depend on the direction of the electric field. In this case, the optimal base for calculation was 1s4l, ml S ≡1s4l, Λ2S+1 . Figure 9 shows the calculated intensities of the $I_{447}$ line in cases where spherical triplet states with l≥2 i Λ=0, 1, 2 and singlet states with l≥2 i Λ=0, 1 were excited.

### 2.4. Measurements and Discussion

The measurement procedure and the analysis of the results consisted of several steps. Firstly, anticrossing spectra were recorded for the defined energies of the ${He}^{+}$ ions. As mentioned, the spectra were recorded in two parts for the positive and negative electric fields. Under single collision conditions, and with a low probability of charge transfer, the intensity of the line was rather weak, and the measurement time had to be sufficiently long. However, maintaining the same experimental conditions over a long time (around 90 h) proved to be extremely difficult. Therefore, measurements were made in multiple 30 min time spans. The electric field was scanned from 0 to ±30 ${kVcm}^{-1}$ with a step of about 50 ${Vcm}^{-1}$. In each step, the photons were counted within 3 s, and the ion beam current was recorded to control the stability of the system’s operation. In this way, by normalising the signal to the ion current, small fluctuations in the experimental system conditions were eliminated.

**Figure 10:**
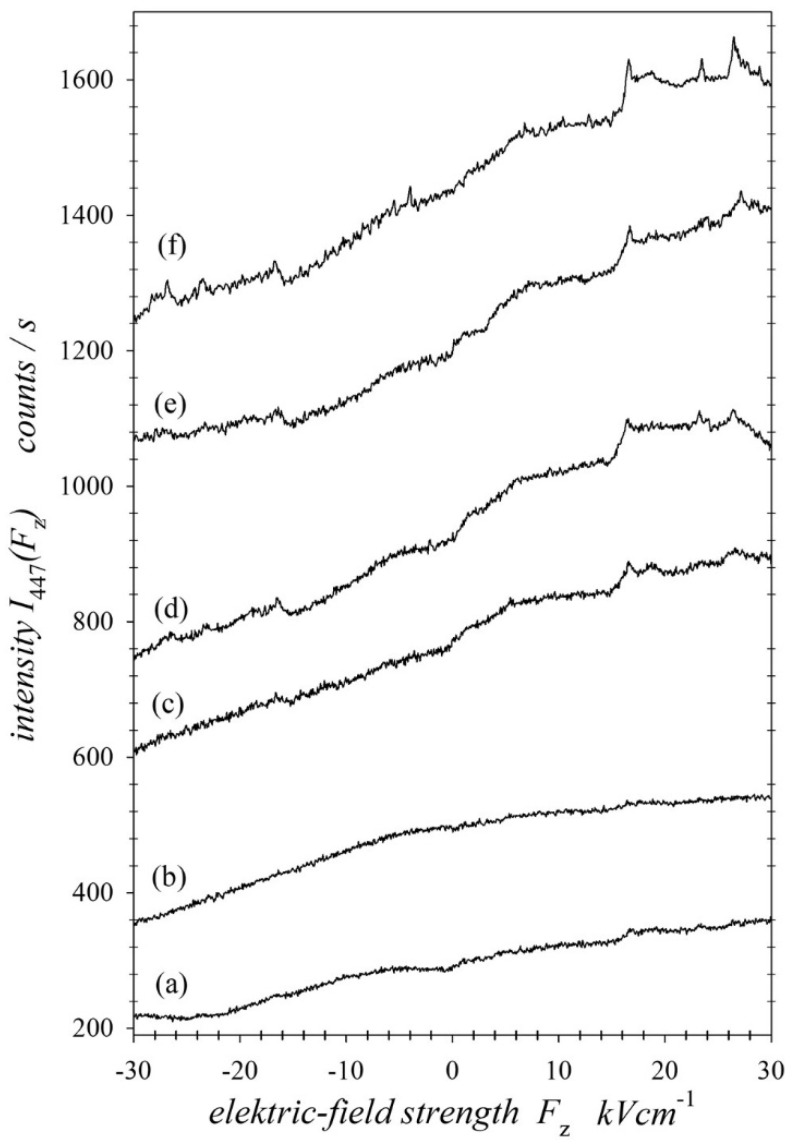
Recorded fluorescence light intensity $I_{447}\left(F_{z}\right)$ of the 447 nm line of HeI, measured as a function of an electric field $F_{z}$ for the charge transfer in collisions ${He}^{+}-He\to {He}^{*}-{He}^{+}$ at impact energies of 10 keV (**a**), 15 keV (**b**), 20 keV (**c**), 23 keV (**d**), 26 keV (**e**), and 29 keV (**f**).

In addition, a control scan was performed every six scans without the target atomic beam to record the background signal, which was then subtracted from the measured signal. Furthermore, the dark count of the optical system without the ion beam was measured before and after the change in the electric field direction. The background signal was less than 50 counts per second, compared with a maximum signal of about 1700 counts per second. In the end, 80–100 scans were added to obtain the final signal. Figure 10 shows the measurement results for ${He}^{+}$ ion energies of 10, 15, 20, 23, 26, and 29 keV. It is evident from the recorded spectra that the charge transfer process strongly depended on the energy of the ${He}^{+}$ ions in the intermediate energy range. The spectra showed an asymmetry to the direction of the electric field, and they could be considered sums of the symmetric and asymmetric components. Therefore, it can be concluded that the excited states of helium atoms after electron capture are a mixture of states with and without the EDM, for which the intensity of the observed line is asymmetric or symmetric to the field direction, respectively. Considering that the Paul trap mechanism is effective in such collisions, we assumed that incoherently excited parabolic Stark states with appropriately directed EDMs were responsible for the asymmetry of the line intensity. In contrast, the symmetric component of the line intensity was due to incoherent excitation of the spherical states, and naturally, parabolic states are the superpositions of spherical states, and vice versa.

**Figure 11:**
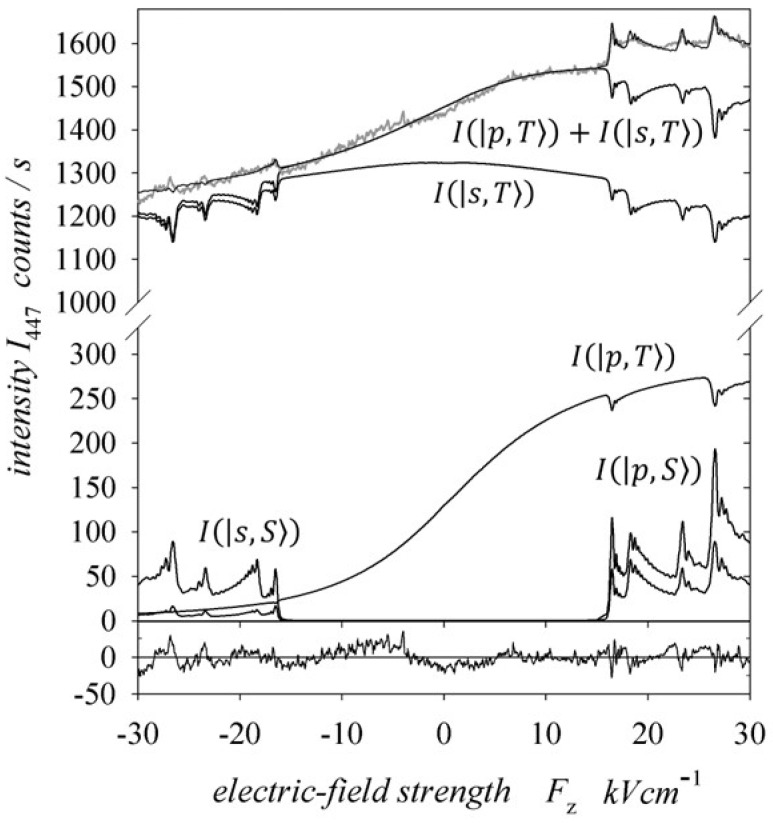
Intensity function $I_{447}\left(F_{z}\right)$ measured for ${He}^{+}(29\;keV)-He\to {He}^{*}-{He}^{+}$ collisions with a theoretically fitted curve and the components $I\left(\left|\left.x,X\right\rangle \right.\right)$ of the intensity originating from the triplet and singlet state excitations X=T,S, respectively (for parabolic and spherical states x=p,s as well, respectively). The solid line at the bottom of the figure illustrates the differences (residues) between the recorded and theoretical signals.

The line λ1s4l D3−1s2p P3=447.2 nm is the result of the transition between triplet states, and thus excited singlet states contributed to its intensity only in the electric field region for which there was an anticrossing of singlet and triplet levels. The excited triplet states contributed to the line intensity over the entire range of the applied electric field. This intensity was greater when the electric field was parallel to the ion velocity and increased as the electric field strength increased. Furthermore, as the collision energy increased, the intensity of the line and the amplitudes of the anticrossing peaks also increased. The increase in these amplitudes means that the relative populations of the anticrossing singlet and triplet levels increased. It is important to note that only one of the three triplet states, with $m_{S}=0,\;\pm 1$, underwent an anticrossing [6]. For energies of 10 and 15 keV, the anticrossing peaks practically disappeared, which means that the populations of the anticrossing singlet and triplet states were equal. Figure 11 shows an example of fitting the theoretically calculated intensities to the spectrum recorded for ${He}^{+}$ ions with an energy of 29 keV. The fit was performed using the least squares method according to the Levenberg–Marquardt algorithm. The theoretical peaks were slightly higher than the experimental ones due to the neglected inhomogeneity of the axial electric field and the discussed velocity distribution of the atoms after electron capture.

The calculated intensities $I_{\lambda }\left(\left|\left.k\right\rangle \right.\right)$ from Figure 8 and Figure 9 were used in the fitting according to the formula

(29) $$I_{\lambda }^{tho}=\sum_{k}{c^{\prime }}_{i_{\lambda }k}I_{\lambda }\left(\left|\left.k\right\rangle \right.\right),\;\;\;\;{c^{\prime }}_{i_{\lambda }k}\geq 0.$$

After normalisation of the fit coefficients $c_{i_{\lambda }k}={c^{\prime }}_{i_{\lambda }k}/\sum_{k}{c^{\prime }}_{i_{\lambda }k}$, we had

(30) $$\sum_{k}c_{i_{\lambda }k}=1$$

The relative cross-sections were calculated, taking into account the degeneracy of the k=k Λs,p1,3 states. Thus, the singlet states k Σs,p1 were not degenerate, the singlet states with Λ=Π,Δ had twofold degeneration, the triplet states kΣs,p3 had threefold degeneration, and the triplet states with Λ=Π,Δ had sixfold degeneration.

Additionally, Figure 11 shows the intensity components $I\left(\left|\left.x,X\right\rangle \right.\right)$ of the observed spectral line. These components are the sum of the intensities coming from the excited triplet and singlet states X=T,S on the basis of the spherical and parabolic states x=s,p, respectively. As previously mentioned, the intensity components originating from spherical states were symmetrical with respect to the electric field’s direction, and the parabolic components showed asymmetry. The solid line at the bottom of Figure 11 illustrates the differences (residues) between the recorded and theoretical signals. The calculated relative cross-sections for charge transfer to excited states for the analysed ion energies are summarised in Table 1. The first row of the table shows the states for which the relative cross-sections $\sigma \left(\left|\left.k\right\rangle \right.\right)$ were determined, with parabolic states in the top row and spherical states in the bottom row. It was estimated that the relative cross-sections for triplet states were determined with a maximum uncertainty below 5%. For the singlet states, which had a much smaller impact on the quality of the fit, this uncertainty did not exceed 20%.

Table 2 collects the percentage contributions of the relative cross-sections σΛs,p1,3 for states with Λ=Σ,Π,Δ for both the spherical and parabolic states. For each excitation energy, these values are given independently for the singlet and triplet states in the top and bottom rows, respectively. In the following columns of the table, the contributions of the cross-sections from all singlet or triplet spherical and parabolic states Ls,p1,3, all spherical and parabolic states $L_{s,p}$, and all singlet and triplet states L1,3 are given. The last column shows the ratio of the relative cross-sections for the triplet states to those for the singlet states L3/L1. Taking into account the normalisation (30), in general, for all analysed collision energies, Table 1 shows that the largest cross-sections for electron capture to singlet states were for the parabolic states 4;0,2,Π1 and 4;0,3,Σ1. Both of these states were characterised by maximal EDMs for the Π and Σ states, respectively. The excitation cross-section of the 4;1,2,Σ1 state with a smaller EDM was larger than that for the 4;0,3,Σ1 state only for a collision energy of 10 keV. However, the singlet parabolic state 4;1,1,Π1, giving a symmetrical contribution to the intensity line (the EDM was equal to zero), had a small cross-section only for the energy higher than 23 keV for the ${He}^{+}$ ion. Interestingly, the excitation of parabolic states is preferred over the entire range of energies considered for singlet states. The share of spherical states 4d,Π1 and 4d,Σ1 increased with the increasing collision energy. For the singlet spherical state 4f,Π1, the cross-section was small, being observed only for collision energies higher than 23 keV. It should be emphasised again that excited singlet Δ states were not recorded in these measurements, but the situation was different in the case of the triplet states. The parabolic state with the largest EDM 4;0,3,Σ3 was not excited at all. As we can see in Figure 8, the calculated spectra for the excited states 4;0,3,Σ3 and 4;0,2,Π3 had steeper slopes compared with the recorded ones in Figure 10. However, the calculated intensities for the excited parabolic triplet states 4;1,2,Σ3 and 4;0,1,Δ3 described the asymmetry of the recorded spectra well. Moreover, the calculated line intensities for these states were close to zero for an electric field of −30 ${kVcm}^{-1}$, which justified the assumption that in the used range of electric field strengths, the triplet parabolic states completely determined the asymmetry of the 447.2 nm line intensity (see the $I\left(\left|\left.p,T\right\rangle \right.\right)$ curve in Figure 11). Obviously, the dominant symmetric part ($I\left(\left|\left.s,T\right\rangle \right.\right)$ curve in Figure 11) of the intensity of the 447.2 nm spectral line came from the incoherently excited triplet spherical states 1s4d, Λ3 and 1s4f, Λ3. As shown in [17,19], for the excited target atoms, the excitation of the parabolic state was not the same as the coherent excitation of the component of this state, containing only the spherical states $\left|\left.1s4d\right\rangle \right.$ and $\left|\left.1s4f\right\rangle \right.$. The 1s4p, Λ3 state was also of great importance, as the influence of the 1s4s, Λ3 state on the intensity of the 447 nm line was negligible.

Moreover, Table 2 shows (the second column from the end) that an increase in the collision energy reduced the parabolic states.

Finally, as can be observed in the last column, the triplet-to-singlet excitation ratio was less than one, which was much lower than the statistical weight of three. However, previous research on the direct excitation of He atoms of a target atom has shown that singlet states are preferentially excited when the Paul trap mechanism operates, particularly in the intermediate energy range [15]. This conclusion is also valid in the case of electron capture by an ${He}^{+}$ ion.

During the collision, a pseudo-molecule ${He}_{2}^{+}$ is created with a core ${\left({He}^{+}\right)}_{2}$ and a promoted saddle electron. During separation, the saddle electron is almost equally likely to remain with the target atom or bind to the projectile ion, thus creating an excited He atom. As already mentioned, the release of saddle electrons and the ionisation of the target atom were also observed. The core ${\left({He}^{+}\right)}_{2}$ of the transient molecule can be in the triplet state Σ3u or singlet state Σ1g, which is essential for the formation of the final singlet and triplet excited state atoms after the projectile and target separate. For a core in the triplet state, the ratio of atoms formed in singlet states to those in triplet states is 3:1. For a core in the singlet state, the opposite is true. The formation of anticrossing peaks means that both excitation paths are realised as a result of collisions at intermediate energies. In [7,15,17,21] and the references contained therein, a detailed discussion of the mechanism of collisions is presented. Table 3 compares the calculated ratios of the cross-sections for the excitation of triplet and singlet states σ4 D3/σ4 D1 resulting from data from [21] and from the current work. In both cases, the estimated uncertainties are reported as standard deviations, and in [21], it was reported that the excitation cross-sections of states 4 D3 and 4 D1 were determined with maximum uncertainties of 15% and 23%, respectively.

An important element of the measurements carried out is the electric dipole moment of the excited states. The density matrix can be used to calculate the EDMs of the excited state mixtures:

(31) $$\langle {\hat{d}}_{z}\rangle =Tr\left(\rho {\hat{d}}_{z}\right).$$

The calculated EDMs are placed in the last column of Table 3. These values can be compared with those for the excitation of the target atoms in ${He}^{+}-He\to {He}^{+}-{He}^{*}$ collisions [15] for n=4 [16] and n=5 [19] and in $He-He\to He-{He}^{*}$ collisions for n=4. However, as expected, the sign of the EDMs in the present measurements was the opposite of that for the excited target atoms. However, in the case of target atoms excited to n=4, the EDM values increased from practically zero to −13.7 $ea_{0}$ [15], and for n=5, they ranged from −7.4±1.6 $ea_{0}$ to −21.1±2.8 $ea_{0}$ [16], with an increase in the collision energy from 10 keV to 29 keV. Meanwhile, in the case of atoms formed as a result of electron capture at n=4 (Table 3), even at 10 keV, the EDM was relatively large (6.1±0.5 $ea_{0}$) and showed slight changes in the range of the applied collision energy up to 29 keV, reaching a maximum (8.5±0.7 $ea_{0}$) for the energy of 15 keV. This is an extremely interesting effect, similar to the observed excitation of target atoms to n=4 in the case of a He−He collision. In this case, the EDM varied from (−5.10±0.22 $ea_{0}$) to (−7.9±0.4 $ea_{0}$), reaching a maximum of (−10.8±0.6 $ea_{0}$) for an energy of 15 keV [19]. The obtained results indicate that in the range of intermediate energies, both the direct excitation process and the electron capture by the ion require further studies. An interesting question is whether in the case of the ${He}^{+}-He$ collision, electrons are also observed as having been scattered forward at a speed greater than the speed of the ion, which was observed in the case of ${He}^{2+}-He\to {He}^{2+}+{He}^{+}+e$ [43]. The mechanism leading to the ejection of such electrons must also be responsible for the distribution of the electron cloud and EDM of excited fast atoms.

## 3. Materials and Methods

The experimental system is based on a linear accelerator that allows ${He}^{+}$ ions to acquire kinetic energy from 10 to 30 keV. This system was previously described in papers [15,16,19,50], and the necessary details for conducting the measurements were provided in Section 2.1. The results were obtained using anticrossing spectroscopy as described in [2,3,4,5,7], with modifications made to the collision chamber, which are detailed in Section 2.1. The measurements were performed for the He4 isotope (purity of 5.0) and provided by the Linde Gas Company.

## 4. Conclusions

In this paper, we determined the post-collisional states with n=4 helium atoms excited in the process of charge transfer in ${He}^{+}-He$ collisions in the range of intermediate energies from 10 keV to 29 keV by measuring and analysing the anticrossing spectra of the λ1s4l D3,F−1s2p P3=447.2 nm line. The experimental results show that the asymmetry of these spectra with respect to the direction of the electric field strongly depended on the ${He}^{+}$ ion energy. For energies below 20 keV, the spectra showed small anticrossing peaks, and for higher energies, the anticrossing resonances became more pronounced. In the post-collision states, the share of parabolic states decreased compared with the spherical states. Although the EDMs of these states, as predicted by the theory, had signs opposite to those of the directly excited target atoms, their values changed slightly in the collision energy range studied. Based on an analysis of the excited states of the target atoms and those resulting from electron capture, it can be inferred that the saddle dynamics and the Paul trap mechanism offer a semiclassical depiction of collisions occurring within intermediate collision energies. Further research is needed to quantitatively interpret and gain a more detailed understanding of the quantum dynamical evolution of bound electron states in the two-centre Coulomb potential of a projectile and target nucleus.

## Figures and Tables

**Table 1:** Relative excitation cross-sections $\sigma \left(\left|\left.x_{i}\right\rangle \right.\right)$ of parabolic (upper row) and spherical (lower row) states for charge transfer excitation of states $\left(n=4\right)$. The estimated uncertainties were about 20% for the singlet states and 5% for the triplet states.

keV	4,0,3,Σ1	4,1,2,Σ1	4,0,2,Π1	4,1,1,Π1	4,0,3,Σ3	4,1,2,Σ3	4,0,2,Π3	4,1,1,Π3	4,0,1,Δ3
	4d,Σ1	4f,Σ1	4d,Π1	4f,Π1	4d,Σ3	4f,Σ3	4d,Π3	4f,Π3	4d,Δ3
10	0.0302	0.2346	0.1493	0.0000	0.0000	0.0049	0.0000	0.0000	0.0139
	0.0000	0.0000	0.0000	0.0000	0.0127	0.0127	0.0278	0.0158	0.0000
15	0.2551	0.0053	0.1420	0.0000	0.0000	0.0000	0.0000	0.0000	0.0127
	0.0000	0.0000	0.0000	0.0000	0.0154	0.0433	0.0274	0.0000	0.0064
20	0.1671	0.0000	0.1509	0.0000	0.0000	0.0132	0.0055	0.0000	0.0033
	0.0342	0.0000	0.0000	0.0000	0.0239	0.0055	0.0137	0.0273	0.0117
23	0.1602	0.0000	0.1177	0.0025	0.0000	0.0233	0.0000	0.0000	0.0042
	0.0578	0.0000	0.0337	0.0000	0.0181	0.0181	0.0198	0.0173	0.0079
26	0.1352	0.0000	0.0954	0.0954	0.0000	0.0060	0.0098	0.0006	0.0006
	0.0292	0.0292	0.0341	0.0341	0.0222	0.0000	0.0044	0.0315	0.0183
29	0.1334	0.0000	0.1006	0.0079	0.0000	0.0107	0.0000	0.0000	0.0053
	0.0608	0.0000	0.0563	0.0000	0.0174	0.0174	0.0173	0.0199	0.0141

**Table 2:** Relative excitation cross-sections σ4Λx1,3 of the spherical (x=s) and parabolic (x=p) states for charge transfer excitation of the states $\left(n=4\right)$ of He atoms. The remaining symbols have the following meanings: Λ1,3—the total contribution from singlet or triplet spherical and parabolic states; Ls,p1,3—the total contribution from all singlet or triplet states of the spherical and parabolic states; L1,3—the total contribution from all singlet or triplet states; and $L_{s,p}$ = the total contribution from all spherical or parabolic states, respectively.

keV	Σp1	Σs1	Σ1	Πp1	Πs1	Π1	Δp1	Δs1	Δ1	Lp1	Ls1	$L_{p}$	L1	L3/L1
	Σp3	Σs3	Σ3	Πp3	Πs3	Π3	Δp3	Δs3	Δ3	Lp3	Ls3	$L_{s}$	L3	
10	0.265	0.000	0.265	0.299	0.000	0.299	0.000	0.000	0.000	0.563	0.000	0.662	0.563	0.78 ±0.11
	0.015	0.076	0.091	0.000	0.262	0.262	0.084	0.000	0.084	0.098	0.338	0.338	0.437	
15	0.260	0.000	0.260	0.284	0.000	0.284	0.000	0.000	0.000	0.544	0.000	0.621	0.544	0.84 ±0.12
	0.000	0.176	0.176	0.000	0.164	0.164	0.076	0.039	0.115	0.076	0.379	0.379	0.456	
20	0.167	0.034	0.201	0.302	0.000	0.302	0.000	0.000	0.000	0.469	0.034	0.561	0.503	0.99 ±0.14
	0.040	0.088	0.128	0.033	0.246	0.279	0.020	0.070	0.090	0.092	0.405	0.439	0.497	
23	0.160	0.058	0.218	0.240	0.067	0.308	0.000	0.000	0.000	0.401	0.125	0.496	0.526	0.90 ±0.13
	0.070	0.108	0.178	0.000	0.223	0.223	0.025	0.047	0.073	0.095	0.379	0.504	0.474	
26	0.135	0.058	0.194	0.194	0.136	0.331	0.000	0.000	0.000	0.330	0.195	0.414	0.524	0.91 ±0.13
	0.018	0.066	0.085	0.062	0.215	0.278	0.004	0.110	0.113	0.084	0.392	0.586	0.476	
29	0.133	0.061	0.194	0.217	0.113	0.330	0.000	0.000	0.000	0.350	0.173	0.414	0.524	0.91 ±0.13
	0.032	0.105	0.137	0.000	0.223	0.223	0.032	0.085	0.116	0.064	0.413	0.586	0.476	

**Table 3:** The ratio σ(4^3^D)/σ(4^1^D) obtained in [21] (the second column) and obtained from our measurements (the fourth column). The EDMs obtained from our measurements in atomic units $ea_{0}$ are presented in the last column.

Obtained in [21]		Obtained in the Present Paper		
Energy/keV	σ4 D3/σ4 D1	Energy/keV	σ4 D3/σ4 D1	EDM
10	1.23 ± 0.47	10	1.16 ± 0.17	6.1 ± 0.5
15	1.87 ± 0.71	15	1.39 ± 0.20	8.5 ± 0.7
20	1.35 ± 051	20	1.15 ± 0.17	7.4 ± 0.6
22.5	1.10 ± 0.42	23	0.89 ± 0.13	6.3 ± 0.5
25	1.05 ± 0.40	26	1.05 ± 0.15	5.6 ± 0.4
30	1.30 ± 0.49	29	0.86 ± 0.12	5.2 ± 0.6

## Data Availability

The datasets used and/or analysed in the current study are available from the corresponding author on reasonable request.
